# Deletion of Constitutive Androstane Receptor Led to Intestinal Alterations and Increased Imidacloprid in Murine Liver

**DOI:** 10.1210/jendso/bvac145

**Published:** 2022-09-21

**Authors:** Anushna Sen, Madison Goforth, Kerry K Cooper, Sayeepriyadarshini Anakk

**Affiliations:** Department of Molecular and Integrative Physiology, University of Illinois at Urbana-Champaign, Urbana, IL 61801, USA; Toxicology Training Program, University of Illinois at Urbana-Champaign, Urbana, IL 61801, USA; Toxicology Training Program, University of Illinois at Urbana-Champaign, Urbana, IL 61801, USA; School of Animal and Comparative Biomedical Sciences, University of Arizona, Tucson, AZ 85721, USA; School of Animal and Comparative Biomedical Sciences, University of Arizona, Tucson, AZ 85721, USA; Department of Molecular and Integrative Physiology, University of Illinois at Urbana-Champaign, Urbana, IL 61801, USA; Toxicology Training Program, University of Illinois at Urbana-Champaign, Urbana, IL 61801, USA; Cancer Center at Illinois, University of Illinois at Urbana-Champaign, Urbana, IL 61801, USA; Division of Nutritional Sciences, University of Illinois at Urbana-Champaign, Urbana, IL 61801, USA

**Keywords:** neonicotinoids, nuclear receptor, imidacloprid, toxicity, gastrointestinal tract, microbiome

## Abstract

Imidacloprid (IMI) is the most frequently detected neonicotinoid pesticide in the environment. Despite typically low toxicity in vertebrates, IMI exposure is associated with liver and gastrointestinal toxicity. The mechanism underlying IMI toxicity in mammals is unclear. Pesticide exposure frequently activates xenobiotic nuclear receptors, such as the constitutive androstane receptor (CAR), to induce detoxification phase I and phase II genes. This study examined the role of CAR in mediating IMI off-target toxicity. Female *Car^−/−^* and wild-type (WT) mice were orally administered imidacloprid (50 mg/kg, twice daily) for 21 days, following which serum, liver, and intestinal tissues were collected. Liver tissue analysis indicated mild inflammation and induction of detoxification gene *Cyp2b10* in IMI-exposed WT mice. The absence of CAR increased hepatic IMI accumulation. Microbiome analysis of ileal samples revealed IMI altered microbial diversity in a genotype-specific manner, with increased α-diversity in *Car^−/−^* mice while decreased α-diversity in WT mice. We observed *Car^−/−^* mice exhibit intestinal alterations with decreased CYP-P450 expression, blunted villi height, and increased small intestine length and weight independent of IMI exposure. Our results suggest that IMI is not overtly toxic. However, the absence of xenobiotic nuclear receptor CAR allows increased accumulation of IMI in the liver and disrupts the villi structure and *Cyp* gene expression in the intestine.

Neonicotinoids account for 25% of the pesticides used worldwide. They are broad-spectrum insecticides with relatively low toxicity in vertebrates [1]. Imidacloprid (IMI) is a routinely used neonicotinoid, and an estimated 1 million pounds is still being used today [2], highlighting the need to understand the consequences of IMI exposure. IMI is predominantly metabolized by oxidative cleavage and hydroxylation in mammals [3], resulting in 6-chloronicotinic acid (6-CNA), 5-hydroxyimidacloprid (5-OH-IMI), olefin derivatives, and desnitro-IMI (formed by reduction via aldehyde oxidase) [4, 5]. Most IMI metabolites are less toxic, except for desnitro-IMI, which is more harmful than the parent compound in mice [6].

Despite being well tolerated, IMI can cause acute poisoning and gastrointestinal symptoms at high doses in humans [7]. Sub-chronic IMI exposure in animal models shows disruption of the gut barrier, induction of liver weight, oxidative stress [8], and cytochrome P450 activity [3, 9, 10]. IMI irreversibly binds to nicotinic acetylcholine receptors in insects, culminating in paralysis and death. But mammalian nicotinic receptors do not bind IMI effectively, and the mechanism for IMI-mediated gastrointestinal toxicity remains unclear. Activation of xenobiotic nuclear receptors (NRs) such as the constitutive androstane receptor (CAR) has been reported with organochlorine (now banned), organophosphate, pyrethroid, and carbamate pesticides [11, 12]. Recently, neonicotinoid thiacloprid was predicted to activate CAR and pregnane X receptor, (PXR) in rats [13]. NRs are ligand-dependent transcription factors that regulate a wide range of physiological processes, including endocrine homeostasis, energy metabolism, and detoxification. Exposure to IMI, a globally used pesticide, has been found to mimic alterations in these processes, resulting in disrupted steroidogenesis, liver metabolism, and obesity [9, 14]. This raises the possibility that IMI exposure may lead to alterations in NR signaling.

In addition to regulating drug/xenobiotic metabolism, CAR can affect endocrine signaling by increasing the clearance of estrogen, progesterone, and thyroid hormone [15]. Further, endocrine signals can target CAR to regulate energy homeostasis; for example, CAR activation is associated with alleviating obesity and diabetes [16, 17]. In fact, chronic dietary pesticide exposure resulted in weight gain in *Car^−/−^* female mice [18]. Due to CAR's detoxification, metabolic and endocrine roles, we investigated if this NR was involved in mediating IMI-induced gastrointestinal toxicity using wild-type (WT) and *Car^−/−^* mice.

## Materials and Methods

### Animal Experiments

Female C57/BL6 mice (#000664) were purchased from Jackson Laboratory. *Car^−/−^* mice were obtained from Dr. David Moore's laboratory as previously described [19]. The mice were housed in flow cages at 24 °C on a 12/12-hour-light/dark cycle, with lights on starting at 6 Am CST, corresponding to zeitgeber time (ZT) 0. Female 8- to 10-week-old mice were used for all experiments. Mice were allowed ad libitum access to a standard chow diet (Teklad Global 18% Protein Rodent Diet, 2918, ENVIGO) and water. WT and *Car^−/−^* mice were randomly divided into a control or treatment group. With oral pipetting, control mice were dosed with vehicle (honey, water, and DMSO mixture, 4:1:1 ratio). Treatment group mice were dosed twice daily (at 7 Am and 7 Pm) for 21 days with 50 mg/kg imidacloprid (PESTANAl, analytical standard, Millipore Sigma) solubilized in a honey-DMSO solvent. Mice were monitored for lethargy, shaking, diarrhea, and body weights were measured once daily over the 21-day treatment period. All mice were sacrificed at ZT4-6. Blood serum and tissues (liver, ileum, colon, kidney, and spleen) were collected for analysis. Tissue was flash-frozen for RNA analysis or fixed in 10% formalin for histological analysis. Genotype was confirmed by PCR (n = 5-8 mice per genotype) as described [19].

### Quantitative Real Time-PCR Analysis

RNA from frozen whole liver tissue and ileum was isolated using TRIzol solution (Ambion) using the manufacturer's protocol. A260/280 ratio and bleach RNA gel were used to determine RNA quality [20]. RNA (3 μg) was DNased (New England Biolabs) and reverse transcribed using random primer mix (New England Biolabs) and the Maxima Reverse Transcriptase kit (Thermo Fisher Scientific). qRT-PCR was performed in triplicates using PerfeCTa SYBR Green FastMix (Quanta). *36b4* and *Ywhaz* were used as housekeeping genes. Primer sequences of genes used are listed in Supplementary Table S1 [21].

### Microbiome Analysis

For analysis, 50 mg of flash-frozen ileal contents were collected per subject, and DNA was extracted using the Qiagen DNeasy PowerSoil Pro kit (Qiagen, Hilden, Germany) per the manufacturer's instructions. The V4-V5 region of the 16S rRNA gene was PCR amplified in triplicate, and pooled barcoded libraries were then sequenced and analyzed with QIIME2 analyzed software (v2020.2) [22–26].

### IMI Quantification

IMI was quantified from 50 µL of serum samples and 50 to 100 mg of frozen liver samples. Frozen livers were powdered using a mortar and pestle on liquid nitrogen. IMI was extracted using 0.5 mL acetonitrile solvent and quantified using mass spectrometry (Metabolomics Center, UIUC).

### Statistical Analysis

All statistical analyses were performed using GraphPad Prism software. Student's unpaired 2-tailed *t* test was used to compare 2 groups. Two-way ANOVA with Bonferroni multiple comparisons test was performed to compare 2 groups with two treatments. Significance was determined by *P* < 0.05. Outliers were determined using Grubbs’ test and removed from the analysis.

### Study Approval

All animal studies were approved by the University of Illinois at Urbana-Champaign Institutional Animal Care and Use Committee.

## Results

### Investigating the Hepatoxicity of Imidacloprid

**Figure 1.**
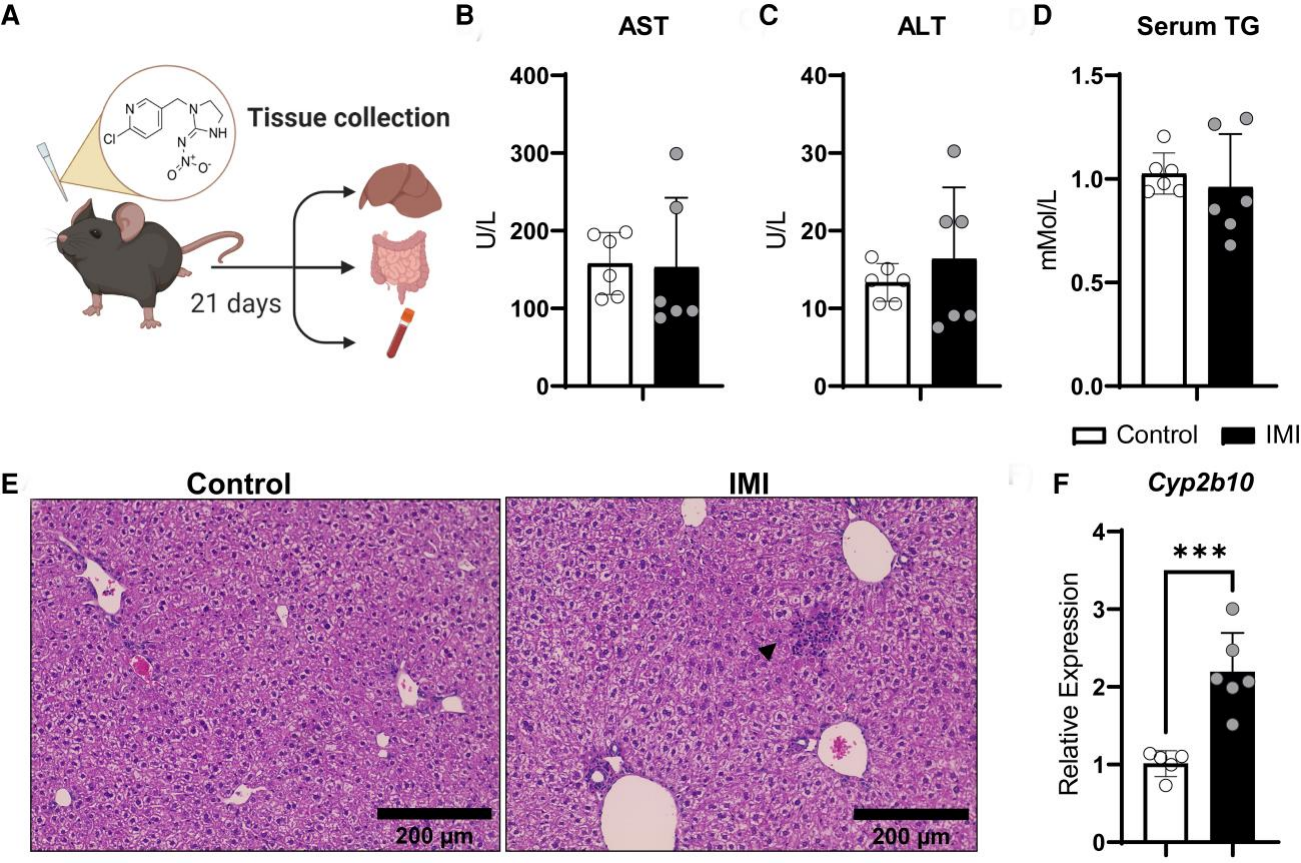
Effect of imidacloprid on the liver. A, Mice were orally dosed with IMI (50 mg/kg BW) twice daily, for 21 days. Tissue samples were collected to examine influence of IMI toxicity. B–D, Serum aspartate transaminase (AST), alanine transaminase (ALT), and triglycerides levels were not induced upon this dose of IMI exposure. E, Representative images of hematoxylin and eosin–stained liver sections from control and IMI-fed mice. IMI-fed livers showed mild inflammatory pockets. F, Detoxification gene *Cyp2b10* was induced in IMI-fed livers. Arrow indicates inflammatory cell cluster. Values are displayed as mean ± SD. Statistics were calculated using Student *t* test analysis. ***P < 0.001. N = 6 mice per group. Schematic was created with BioRender.com.

The liver is the primary organ for detoxification, and previous studies have reported hepatic IMI accumulation and hepatotoxicity [27]. To mimic the most likely exposure route in humans, mice were orally fed IMI mixed into a honey solvent (Fig. 1A). We selected a sub-acute period of 21 days of dosing, since liver toxicity is reported between 15 to 28 days for mice fed 15 to 20 mg/kg IMI [9, 28]. We selected a 50 mg/kg IMI twice daily dosage, comparable to the estimated ingestion amount in a retrospective cohort study that exhibited gastrointestinal symptoms upon IMI ingestion [7]. Serum levels of aspartate transaminase (AST) and alanine transaminase (ALT) are often induced upon liver injury, but IMI exposure did not induce these markers (Fig. 1B and 1C). Serum triglycerides also remained unaffected (Fig. 1D). Next, we examined the liver histology using hematoxylin and eosin staining (Fig. 1E) but did not observe overt hepatocellular changes [29]. However, IMI exposure resulted in pockets of inflammatory cells in the hepatic lobules and mild pericholangitis. To examine if CAR is activated in response to IMI, we analyzed the expression of *Cyp2b10,* a bona fide transcriptional target of CAR (NR1I3). We found that IMI treatment led to the induction of *Cyp2b10* (Fig. 1F). Apart from mild focal inflammation and increased *Cyp2b10* gene expression, IMI did not lead to hepatoxicity in mice after 21 days of exposure.

### Deletion of CAR Results in Accumulation of Hepatic IMI Concentration, Not Hepatotoxicity

**Figure 2.**
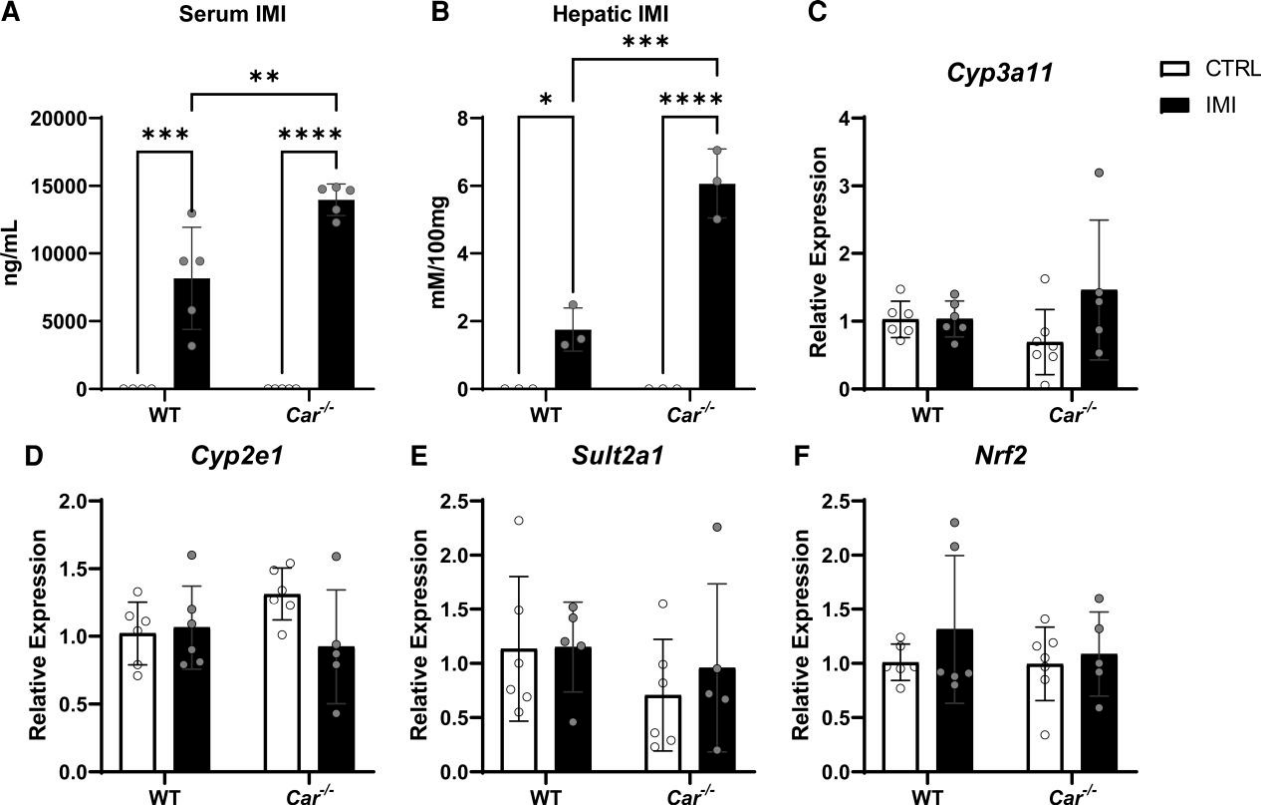
Excess IMI accumulation in the *Car*^−/−^ liver does not alter hepatic Cyp450 gene expression. Serum and liver tissue was analyzed by mass spectrometry to measure IMI accumulation. *Car^−/−^* mice accumulated more IMI than wild-type mice (A-B). *Car^−/−^* and wild-type livers (control and IMI-fed) were examined to quantify expression of genes involved in detoxification and oxidative stress. Hepatic gene expression of phase I genes *Cyp3a11* (C), *Cyp2e1* (D), and phase II *Sult2a1* (E) and antioxidant regulating transcription factor *Nrf2* (F) was unaltered by IMI exposure. Values are displayed as mean ± SD. Statistics were calculated using two-way ANOVA with Bonferroni post hoc analysis. *P < 0.05, ***P < 0.001, ****P < 0.0001. N = 5-8 mice per group.

*Car^−/−^* and WT mice were exposed to IMI for 21 days, and the levels of IMI in serum and the liver were measured using LC-MS. As expected, vehicle-treated WT and *Car^−/−^* mice displayed no detectable levels of IMI. Intriguingly, *Car^−/−^* mice accumulated 1.7-fold more IMI in the serum, and 3-fold more IMI in the liver than WT mice (Fig. 2A and 2B), suggesting that CAR is necessary to control both the circulating and hepatic IMI concentrations.

A frequent adverse effect of pesticide exposure is weight gain [30]. Therefore, we monitored daily body weight changes as percent difference of original body weight (Supplementary Fig. S1A [21]) after IMI exposure. IMI exposure did not show any significant difference in weight gain in either WT or *Car^−/−^* mice (Table 1). We also analyzed and found no change in the expression of *Car* transcript in the liver, ileum, or kidney, all organs involved in IMI uptake, metabolism, and clearance (Supplementary Fig. S1B [21]).

**Table 1.** Gross organ and body weight measurements of WT and *Car^−/−^* mice treated either with vehicle or IMI

Organ	WT vehicle	WT IMI	*Car^−/−^* vehicle	*Car^−/−^* IMI
**Body weight (g)**	21.27 ± 0.7	20.65 ± 0.78	19.41 ± 1.9	19.3 ± 0.66
**Liver weight (g) to body weight (g) %**	4.52 ± 0.3	4.55 ± 0.3	4.34 ± 0.2	4.57 ± 0.4
**SI weight (g) to body weight (g) %**	3.97 ± 0.1	3.72 ± 0.7	5.66 ± 0.9**	5.22 ± 0.89*
**SI length (cm) to body weight (g) %**	152.9 ± 6.4	159.5 ± 4.4	192.1 ± 12.4****	197.4 ± 5.3****
**Colon weight (g) to body weight (g) %**	1.3 ± 0.2	1.3 ± 0.3	1.48 ± 0.2	1.49 ± 0.4
**Colon length (cm) to body weight (g) %**	32.94 ± 1.7	35.13 ± 2.1	37.03 ± 3.6	36.91 ± 3.8
**Kidney weight (g) to body weight (g) %**	1.13 ± 0.04	1.17 ± 0.02	1.41 ± 0.09	1.07 ± 0.07
**Spleen weight (g) to body weight (g) %**	0.31 ± 0.01	0.34 ± 0.04	0.4 ± 0.1	0.31 ± 0.07

Values are displayed as mean ± SD. N = 5-8 mice per group. Statistics were calculated using two-way ANOVA with Bonferroni post hoc analysis. Abbreviations: IMI, imidacloprid; SI, small intestine; WT, wild-type. *P < 0.05, **P < 0.001, ****P < 0.0001, *Car^−/−^* control with respect to WT control and *Car^−/−^* IMI with respect to WT IMI.

### *Car^−/−^* Mice Have Altered Small Intestine Morphology

Since IMI has been reported to have multi-organ adverse effects, organ weights (liver, small intestine, colon, kidney, and spleen) were analyzed with respect to the body weights (Table 1). The kidney and spleen were examined for toxicity as they are additional sites of IMI accumulation [31, 32]. Kidney and spleen weights were unaltered, although *Car^−/−^* mice exposed to IMI had a decreasing trend compared to vehicle control (Table 1). Kidney and spleen histological features were comparable between treatment and genotype (Supplementary Fig. S2 [21]). Neither treatment nor genotype significantly altered hepatosomatic index (liver to body weight ratio). Surprisingly, despite accumulating more IMI, *Car^−/−^* livers did not show an increase in inflammation but rather were milder than WT mice (Supplementary Fig. S1D [21]).

Absorption in the gut is important for orally ingested pesticides to exert potential toxicity. IMI is rapidly taken up by intestinal transporters [33] and can also disrupt the colon's tight junctions, resulting in increased intestinal permeability and decreased ileal bile acid transporter gene expression [8, 34]. Thus, to assess the role of CAR in IMI-mediated intestinal toxicity, both the small and large intestine (along with luminal contents) were collected from WT and *Car^−/−^* mice. While we did not observe any intestinal or colon dysfunction post-IMI, intriguingly, loss of CAR was sufficient to increase small intestine weight and length significantly (Table 1).

### Intestinal Expression of *Cyp* Genes and Ileal Structure Were Lost in *Car^−/−^* Mice

IMI toxicity can be attributed to increased reactive oxygen species generation, inflammation, and toxic metabolite formation. To test if these mechanisms were employed in *Car^−/−^* mice, quantitative polymerase chain reaction (PCR) analysis of liver and ileal gene expression were performed.

Hepatic metabolism can be a double-edged sword; while it is crucial for detoxification and clearance of pesticides, it can also produce toxic metabolites. Mammalian metabolism of IMI occurs predominantly by oxidative cleavage and hydroxylation [3], forming IMI metabolites such as 6-chloronicotinic acid, 5-OH-IMI, olefin derivatives, and, desnitro-IMI (formed by reduction via aldehyde oxidase) [4, 5]. In particular, human cytochrome P450 3A4 [35] is important for IMI metabolism.

Because CAR is known to transcriptionally control several members of the CYP P450 family and aldehyde oxidase [36], we examined if IMI metabolism is altered in *Car^−/−^* mice. We measured *Cyp3a11* expression, the mouse homolog of human CYP3A4 (responsible for imidazolidine oxidation *in vitro*) (Fig. 2C) [3], *Cyp2e1,* responsible for nitroimine reduction (Fig. 2D), and phase II *Sult2a1* because increased sulfotransferase expression has been reported in IMI resistant insects (Fig. 2E) [37]. In addition to their previously reported roles in IMI metabolism, these genes are also *bona fide* targets of CAR. In contrast to previous studies in lizards and fish [38, 39], IMI did not induce xenobiotic-metabolizing phase I genes in either WT or *Car^−/−^* livers (Fig. 2).

Furthermore, several genes involved in phase II conjugation (*Gsta1, Gstm2, Gstp1,* and *Sult1e1*), oxidative stress (*Nqo1, Aox1, Akr*), and proliferation (*Cyclin D1, Cyclin B1*) (Supplementary Fig. S3A-S3J [21]) remained unaffected by IMI exposure.

**Figure 3.**
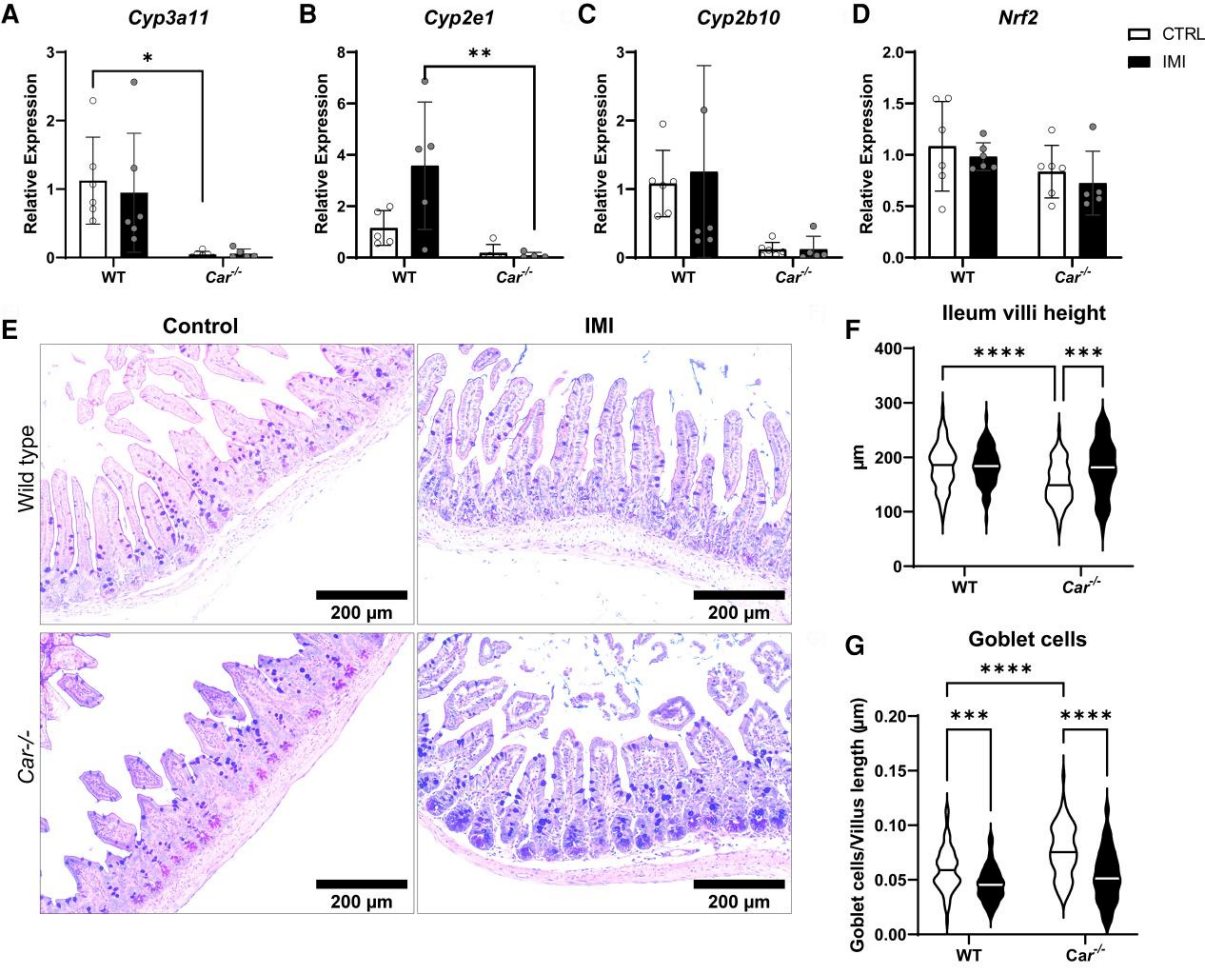
Absence of CAR drastically reduced ileal Cyp450 gene expression and alters villi length while IMI reduces mucus production in the ileum. Ileal tissues from *Car^−/−^* and wild-type mice (control and IMI-fed) were examined to quantify expression of genes involved in detoxification and oxidative stress. Ileal expression of phase I genes *Cyp3a11* (A), *Cyp2e1* (B), *Cyp2b10* (C), were unaltered by IMI exposure. However, *Cyp3a11* (A) and *Cyp2e1* (B) expression, was blunted in *Car^−/−^* ileum. Expression of *Nrf2 gene,* which is a key regulator of antioxidant mechanismswas also unaltered by IMI exposure (D). Values are displayed as mean ± SD. N = 5-8 mice per group. Ileum tissue sections were analyzed for villi structure and mucin production by Alcian Blue/Periodic Acid-stain (E). *Car^−/−^* villi had decreased length compared to wild-type control (F). Goblet cells were decreased in IMI-treated wild-type and *Car^−/−^* mice compared to their respective control group (G). Statistics were calculated using two-way ANOVA with Bonferroni post hoc analysis. *P < 0.05, **P < 0.01. N = 5 mice per group, 15 villi per mouse. 200 μm.

Since the absence of CAR resulted in morphological changes in the small intestine, we examined the expression of *Cyps* and other metabolic genes. We chose to study the ileum as IMI was shown to reduce ileal gene expression. Surprisingly, *Car^−/−^* ileum displayed significantly blunted *Cyp* expression than WT mice, independent of IMI exposure (Fig. 3A-3C).

Next, we examined IMI's role in hepatic and ileal reactive oxygen species generation. Transcription factor *Nrf2* regulates the expression of antioxidant proteins during oxidative stress [40]. IMI exposure did not alter *Nrf2* expression in either group, suggesting an absence of oxidative stress (Figs. 2F and 3D).

Since *Car^−/−^* mice ileum showed contrasting features with WT mice, we examined the intestinal villi, the functional unit for digestion, absorption, and secretion in the intestine (Fig. 3E). Villi height and shape can be regulated by dietary factors, and toxins [41]. Villi height can also be under NR control; PPAR activation can increase villi height, whereas mutated TRα in mice decreases villi size [42, 43]. We found that IMI increased villi height in *Car^−/−^* mice but not WT mice (Fig. 3F). Goblet cells in the ileum produce acidic and neutral mucus [44]. IMI decreased mucus production in both WT and *Car^−/−^* mice (Fig. 3G).

### IMI Influence on Ileum Microbiome

**Figure 4.**
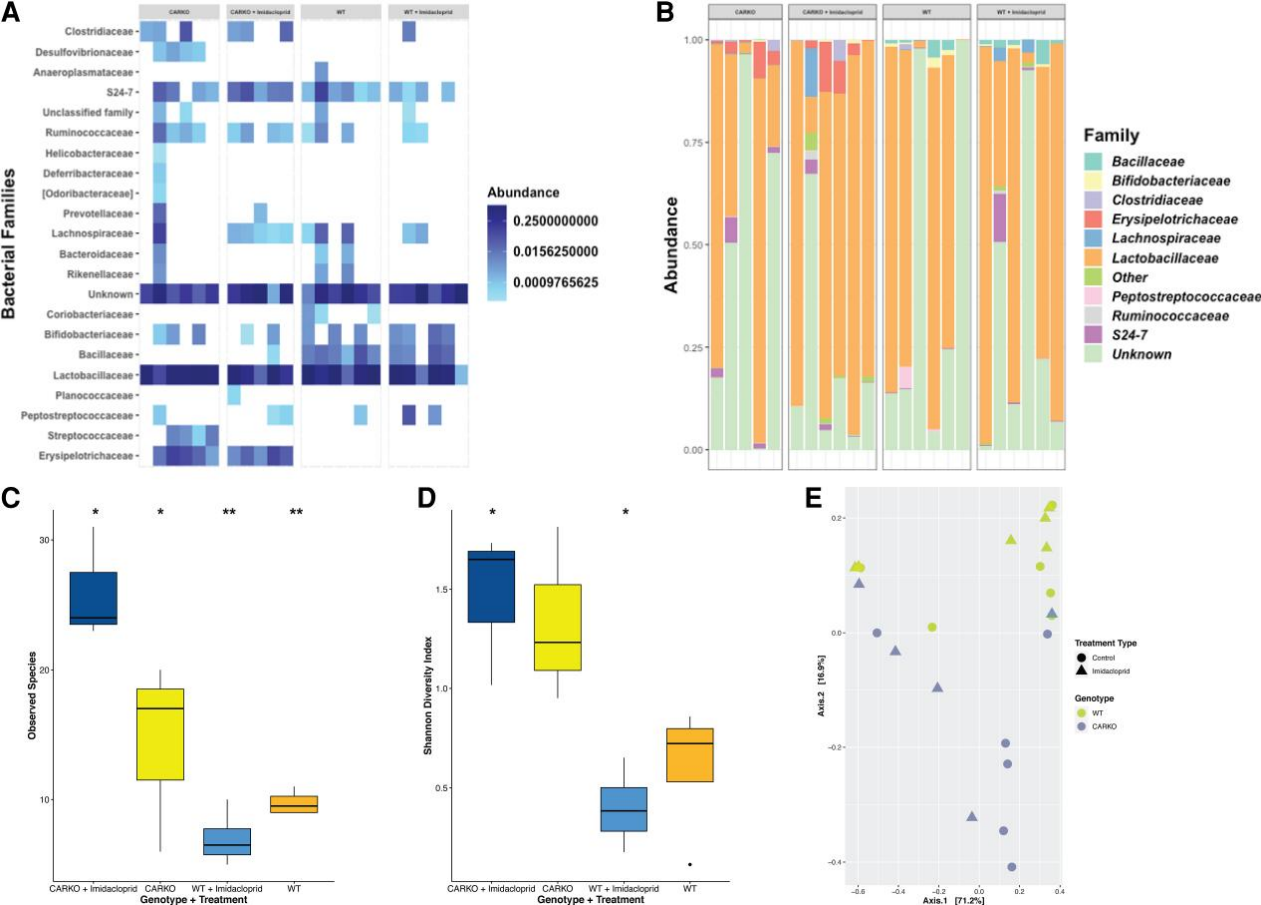
Influence of Imidacloprid on ileum microbiota. Abundance heat map of bacterial families varied more by the genotype than treatment (A). Taxonomic abundance plots showed *Erysipelotrichaceae* spp more prevalent in *Car^−/−^*, while *Bacillaceae* spp were present in higher levels in WT (B). Number of observed species based on amplicon sequence variant (ASV) present in each type of treatment (C). Shannon diversity (evenness and distribution) of bacterial species (D). Bray Curtis PCoA plot shows bacterial communities clustered more by genotype than treatment (E).

The gut microbiome has been shown to be critical in modulating gut barrier function through numerous mechanisms [45]. Therefore we wanted to investigate if IMI altered the ileal microbiome as another potential mechanism for impacting gut barrier function. *Car^−/−^* mice had higher levels and/or were more consistently colonized with *Clostridiaceae* and *Erysipelotrichaceae* compared with the WT mice, regardless of treatment status (Fig. 4A and 4B). However, the untreated *Car^−/−^* mice had more prevalence of *Desulfovibrionaceae* and *Streptococcaceae* compared to IMI-treated *Car^−/−^* mice or WT mice with or without treatment, where it was either absent or present at significantly lower levels. Interestingly, IMI treatment of *Car^−/−^* mice increased the prevalence of *Lachnospiraceae*, but this was not observed in WT mice. Whereas WT mice had a higher prevalence of *Bifidobacteriaceae* and *Bacillaceae* compared to *Car^−/−^* mice, neither family was heavily impacted by treatment. In fact, there did not seem to be any major shifts in the prevalence of ileal bacterial families in WT mice due to IMI treatment (Fig. 4A and 4B). The core bacterial members were determined at a prevalence of 0.01% and an abundance of 0.01% for each of the genotypes, treatment groups, and across all 4 groups, and it was found that *Lactobacillaceae* were present in all 4 groups as part of an overall core ileal microbiome. Interestingly, treatment with IMI resulted in a statistically significant increase in the number of observed species in *Car^−/−^* mice (*P* value 0.01). In contrast, there was a statistically significant reduction in the observed species in IMI-treated WT mice compared to the WT control (*P* value 0.01) (Fig. 4C). Similarly, there was a significant increase in the Shannon diversity in *Car^−/−^* mice after IMI treatment (*P* value 0.01), while the Shannon diversity decreased in the WT mice after IMI exposure (*P* value 0.01) (Fig. 4D). Examining the beta diversity of the samples found that the samples did not cluster tightly together based on any one variable, which suggests a large amount of variation in the microbiome composition among all the mice. However, the samples did separate based on the WT genotype vs *Car^−/−^* mice, but these samples did not cluster based on IMI for either genotype (Fig. 4E). Based on these results, IMI does not appear to cause large shifts in the composition of the ileal microbiome overall for either *Car^−/−^* or WT mice but instead impacts a few bacterial families to the point that those community members almost or do disappear entirely. These bacterial community shifts are enough to alter the overall diversity of the ileal microbiome, but the impact depends on the initial starting microbiome composition.

## Discussion

Imidacloprid (IMI), the first neonicotinoid pesticide, has become the pesticide of choice for agriculture, infrastructure, and pets. Neonicotinoids are detectable in the environment, and IMI had the highest frequency of occurrence, present in over 50% of the collected samples of honey [46]. Data from the National Health and Nutrition Examination Survey (NHANES) indicate that IMI and its metabolite 5-OH-IMI are detectable in the USA general population. IMI metabolites had a higher detection frequency and concentration than the parent compound [47, 48]. We investigated the role of the xenobiotic NR CAR, since CYP450 activation, altered metabolism, and hepatocyte hypertrophy noted upon IMI exposure are all cellular processes known to be regulated by CAR [44, 45]. The liver and small intestine are two major metabolic sites for xenobiotic transformation as well as for the expression of CAR [49, 50], which coordinates the transcription of phase I and phase II detoxification genes.

IMI exposure is associated with altered metabolism, obesity, and gut barrier disruption [8, 9, 14], and CAR function is linked with these cellular outcomes. Therefore, to test if IMI-mediated gastrointestinal defects were CAR-driven, we orally dosed control and *Car^−/−^* mice with IMI.

Consistent with earlier in vitro studies, where human hepatic CYP2B6 was found to metabolize IMI [3], we found that mouse homolog, *Cyp2b10* was induced in murine livers upon IMI exposure. Importantly, CAR deletion led to a dramatic decrease in *Cyp2b10* gene expression that correlated well with an increase in serum and hepatic IMI accumulation.

Despite an increase in hepatic IMI content in *Car^−/−^* mice, we do not observe obvious hepatotoxicity, which contrasts with previous studies that showed a mild increase in serum liver injury markers [9, 28]. A retrospective IMI case study also revealed that 4 patients (out of 128) developed delayed liver injury [51]. The dose, timing, oral delivery, and vehicle may contribute to this discrepancy. It is possible that using honey, a rich source of antioxidant polyphenolic acids [52, 53], albeit routinely used as a delivery vehicle [54], may alleviate some toxic effects. Polyphenols protect against pesticide toxicity [55], but we do not think this is the case since we used a small dose of honey (2 g/kg body weight) [56]. On the other hand, in a mouse cancer study, despite IMI being administered at a much higher dose of up to 208 mg/kg/day and 274 mg/kg/day for 2 years (MRIDs 42256335 and 42256336) [57], no changes in serum chemistry, gross or histopathology were reported. These data, including our findings, indicate that IMI is well tolerated by the liver. To reconcile the conflicting findings [8, 14, 28, 58], we posit that more likely additional hits, like diet-induced obesity [14], may elicit a toxic response to IMI exposure.

We then investigated IMI's effect on the gastrointestinal tract by analyzing the liver and the intestinal CYP450 gene expression and the ileal gut microbiome. Previously, IMI exposure was shown to alter the intestinal barrier and the colon microbiome [8]. Few studies have examined CAR and gut microbiome interaction. One study suggested that the activity and expression of CAR in the colon can be altered by the gut microbiota [59], and its expression is linked to mucosal homeostasis (colon), cecal and fecal microbiome diversity, and basal expression of certain intestinal CYPs [50, 59–62].

In this paper, apart from the liver, we also focused on the ileum. Compared with WT mice, we found an increase in length and weight of the small intestine but shortened villi in the ileum, changes in P450 gene expression, and the ileal microbiome in *Car^−/−^* mice. Of note, variation in small intestine length is linked with either adaptation to nutrient availability or intestinal disease [63].

CYP expression and metabolism of certain drugs (phenacetin and flurazepam) have been previously identified in the gut [64]. *Car^−/−^* ileum displayed low expression of *Cyp2e1*, which, when induced, has been shown to increase gut leakiness [65]. Although we observed a trend for *Cyp2e1* induction in IMI-treated WT ileum, it was not statistically significant. We also observed a reduction in mucous-secreting cells with Alcian blue staining upon IMI exposure in WT and *Car^−/−^* ileum, indicative of a possible gut barrier dysfunction.

Concomitantly, we found that IMI modified abundance and microbiome diversity in a genotype-specific manner, with an increase in *Car^−/−^* but a decrease in WT female ileum. In contrast, male mice fed IMI for 70 days had increased colon microbial diversity [8]. The differences in the duration of IMI treatment, sex, and the intestine region could contribute to the observed discrepancy. Importantly, our finding of a higher abundance of the ileal microbiome aligns with recent results from cecal and fecal *Car^−/−^* samples [61, 62]. *Car^−/−^* ileum also had increased microbiome diversity. The absence of CAR expression can increase pro-inflammatory bacteria [62]. We find bacteria *Erysipelotrichaceae*, positively associated with inflammatory mouse models [66], increase uniquely in *Car^−/−^* ileum. When examining the effect of environmental contaminants on bacterial abundance via NR modulation, a study found that Aroclor1260-exposed *Car^−/−^* mice on a high-fat diet had higher levels of the *Lachnospiraceae* family. IMI-exposed *Car^−/−^* mice exhibited a similar increase in *Lachnospiraceae* [61]. *Streptococcaceae* and *Desulfovibrionaceae* had increased abundance in control *Car^−/−^* ileum but were absent upon IMI exposure. The decrease in *Desulfovibrionaceae* upon neonicotinoid exposure has been previously reported [67]. Gut dysbiosis is associated with liver disease. For example, patients with nonalcoholic fatty liver disease have a microbiome enriched with *Lachnospiracea, Erysipelotrichaceae*, and *Streptococcaceae* [68]. Thus, altered gut microbiota observed in IMI-exposed and control *Car^−/−^* mice may increase susceptibility to liver diseases.

Some limitations of this study include using a single dose of IMI and only studying females. The rationale for selecting female mice is that the detection of IMI is higher in females than males [69], and there is increasing evidence of adverse effects of IMI on female reproductive toxicity; for instance, female rats exposed to IMI for 90 days had serum hormonal changes with increased follicle-stimulating hormone (FSH), decreased luteinizing hormone (LH), and progesterone levels [70]. In addition, CAR activation is more robust in female mice. But future studies must include both sexes in order to determine if there is a sex-specific response to IMI.

In conclusion, we find the dose of IMI we tested is not overtly toxic. Nevertheless, we uncovered an ileal role of CAR in maintaining gut function and microbiota. Reduced P450 expression in *Car^−/−^* ileum could indicate reduced metabolic capacity and increased susceptibility to xenobiotics. However further studies are necessary to understand the intestinal role of CAR fully.

## Data Availability

Microbiome sequence data is deposited on GenBank as BioProject accession number: PRJNA826115. https://www.ncbi.nlm.nih.gov/sra/PRJNA826115.

## Abbreviations

CAR: constitutive androstane receptor
IMI: imidacloprid
NR: nuclear receptor
PCR: polymerase chain reaction
PXR: pregnane X receptor
WT: wild-type
